# Design and Validation of a Custom-Made Hyperspectral Microscope Imaging System for Biomedical Applications

**DOI:** 10.3390/s23052374

**Published:** 2023-02-21

**Authors:** Jošt Stergar, Rok Hren, Matija Milanič

**Affiliations:** 1 Jožef Stefan Institute, Jamova Cesta 39, SI-1000 Ljubljana, Slovenia; 2 Faculty of Mathematics and Physics, University of Ljubljana, Jadranska ulica 19, SI-1000 Ljubljana, Slovenia

**Keywords:** biomedical optics, microscopy, hyperspectral imaging, spectroscopy, instrumentation

## Abstract

Hyperspectral microscope imaging (HMI) is an emerging modality that integrates spatial information collected by standard laboratory microscopy and the spectral-based contrast obtained by hyperspectral imaging and may be instrumental in establishing novel quantitative diagnostic methodologies, particularly in histopathology. Further expansion of HMI capabilities hinges upon the modularity and versatility of systems and their proper standardization. In this report, we describe the design, calibration, characterization, and validation of the custom-made laboratory HMI system based on a Zeiss Axiotron fully motorized microscope and a custom-developed Czerny-Turner-type monochromator. For these important steps, we rely on a previously designed calibration protocol. Validation of the system demonstrates a performance comparable to classic spectrometry laboratory systems. We further demonstrate validation against a laboratory hyperspectral imaging system for macroscopic samples, enabling future comparison of spectral imaging results across length scales. An example of the utility of our custom-made HMI system on a standard hematoxylin and eosin-stained histology slide is also shown.

## 1. Introduction

Established histopathology relies on color and shape information collected by standard laboratory microscopy, typically using stained tissue cell specimens. One of the ways to offer new insights into histopathologic diagnosis is to add spectral-based contrast by means of hyperspectral imaging (HSI), which offers the advantages of quantitative absorbance measurements and computation of contrast resulting from light scattering on tissue constituents. Such an integrated approach, often called hyperspectral microscope imaging (HMI) [1], has already been used in the biomedical field to detect various types of carcinoma and other pathological conditions [2,3,4,5,6,7] and may be instrumental in establishing quantitative histopathology as the new diagnostic standard.

Different approaches to HMI have been used in the past, including point-wise scanning [8,9], linear push-broom scanning [6,10], snapshot mapping [5,11,12], filtered techniques based on liquid crystal tunable filters (LCTF) [13], and acousto-optic tunable filters (AOTF) [14,15], either on the emission or excitation side. Although such systems offer a broad spectral range in hundreds of nanometers, their spectral resolution is typically limited to a few 10 nm [16,17]. In the literature, the performance of methods has also been improved by compressive sensing, implemented through digital micromirror device (DMD) encoding [18] or through the use of coded-aperture masks [19,20]. As shown by recent studies [21], rapid development of different HMI modalities and applications has been achieved in the last few years. The different systems, however, follow different characterization protocols, which makes standardization problematic. One of the motivations of the present paper is to present a template of metrics to be evaluated as part of the standardization. Furthermore, due to the relatively large price of hyperspectral microscopy systems, a reduction in price is needed to facilitate the development of the field. The system presented in this paper strives to be applicable to a broad range of different existing microscopes through its modular design, thus decreasing the price of setting up a hyperspectral microscope. The spectral filtering approach enables selectivity in spectral ranges to be imaged, a feature that is usually absent in spatial scanning methods (push-broom and point-wise scanning). This spectral selectivity can, in turn, decrease the acquisition time and can further be used to test more multispectral acquisition schemes without any additional modification.

Hyperspectral microscopy has found applications within the biomedical [21] and other fields, such as nanotechnology [22]. Many biomedical studies focus on color enhancements, digital staining, and fluorescence imaging [21]. One promising option frequently used in the analysis of hyperspectral microscopy images is the use of artificial intelligence for segmentation and classification [23,24]. A logical next step is the quantitative analysis of chromophore concentration, with possible applications in the development of quantitative imaging and laying a foundation for the development of quantitative imaging biomarkers. This development, however, requires the use of calibrated and characterized microscopy systems that can reliably measure both optical and dimension-related properties of the samples.

The primary objective of this study was to validate the custom-made HMI system with the overall goal of piloting standardization of the protocols. While designing a custom-made HMI system is demanding, systematic application of validation protocols offers grounds for future standardization, which may also influence the development of commercial systems. Furthermore, the performance of the developed system was compared and validated against the hyperspectral system for macroscopic sample imaging.

Achieving the objectives thus put forth is especially necessary to ensure the repeatability of measurements using the system and to enable quantitative imaging. To achieve these goals, the presented protocol characterizes the most important parameters of a hyperspectral microscope imaging system as well as verifies it against a reference method. In turn, the imaging system presented in this paper is, to the best of our knowledge, the first that is characterized, verified against a reference method, and compared to a hyperspectral imaging system for macroscopic imaging. Additionally, the comparison of results against the system for macroscopic imaging offers new possibilities for reliably imaging samples across different length scales, thus addressing an open question in biophotonics [25].

In the first section of the paper, we describe the design of the system; in the second section, we discuss its calibration and characterization; and finally, we show the system’s validation and an example of its use in biomedicine.

## 2. Materials and Methods

This section of the paper describes the development and construction of the HMI system, starting with the system requirements and following through with a detailed description of system components. It concludes with an adaptation of the previously published validation and calibration protocol [26] applicable to the filtered methodology of hyperspectral imaging

### 2.1. HMI System

Before constructing the system, the following requirements for the HMI system were identified to enable the imaging of biological samples and facilitate further quantitative analysis:

1. The system should offer a spectral range from approximately 400 nm to 800 nm (dictated by chromophores) with a spectral resolution in the range of 1 nm–5 nm (to study fine spectral features of chromophore absorption).
2. The spatial resolution should be greater than 5 µm to discern changes inside the cell nucleus.
3. The wavelength range should be adjustable to decrease sample acquisition time; automatic acquisition of multiple areas on the sample should be possible to enable imaging of large samples.
4. The system should be modular, allowing for any pertinent modifications in the future, and should also be adaptable to different microscopes.
5. Since samples were expected to be thin, the primary modality was transmission imaging.

The selection of the wavelength range between 400 nm and 800 nm is based on four important technological and physical considerations. (A) The majority of commonly studied tissue native chromophores have features in this range (e.g., hemoglobin, beta carotene) [27]. (B) Histopathological slides are stained with stains that are optimized for visible light contrast due to the traditional nature of observation with the naked eye through the microscope. (C) Many commercial systems have optics designed to perform best in the visible part of the spectrum. This choice of the wavelength range thus makes the system applicable to a broader range of microscopes. (D) Quantum efficiency of most nonspecialized silicon-based detectors decreases at wavelengths higher than 800 nm.

In the continuation, we briefly outline the major components of the system with additional technical information outlined in Appendix A and Appendix B.

#### 2.1.1. Microscope

The core system is based on a Zeiss Axiotron (Carl Zeiss, Germany) upright inspection microscope equipped with an auto-focus system (Carl Zeiss, Germany) that is used for computerized control of the focusing mechanism (z-axis manipulation). It is further upgraded with a motorized stage and controller for movement in x–y directions (MC2000, Marzhauser-Wetzlar, Wetzlar, Germany). Together, these upgrades enable full computerized control of positioning and refocusing, thus fulfilling the requirement for whole slide imaging. The microscope is fitted with Epiplan-NEOFLUAR HD objective lenses (2.5×, 5×, 10×, 20×, 50×, 100×). These objective lenses are optimized for imaging without cover glass, but according to the manufacturer’s specifications, lenses up to 20× can also be used with samples sporting coverslips without unacceptable deterioration in image quality. A specific advantage of the Epiplan-NEOFLUAR HD series is the presence of a darkfield illumination ring, which can be used in future research.

#### 2.1.2. Camera

For image acquisition, a high-quality camera is required with the following specifications:

1. Due to the low amount of light per spectral band and, thus, longer exposure times, both low dark current and high quantum efficiency of the detector are required.
2. The light intensity can vary dramatically in the specified spectral range; therefore, a high well depth resulting in a large dynamic contrast is needed.
3. The resolution of the camera must be good enough to provide detailed images of the samples.

To satisfy these conditions, the microscope is coupled to an Andor Zyla 5.5 USB3.0 camera (Oxford Instruments, Belfast, UK), which has a resolution of 5.5 megapixels (image size 2560 × 2160), 16.6 × 14.0 mm active area, 64% quantum efficiency at 600 nm as well as low readout noise (0.9 e^−^ at 200 MHz readout rate) and exceptionally low dark current (0.1 e^−^/pixel/s with forced air cooling). The full dynamic range employing 16-bit architecture is 33,000:1. The pixel size is 6.5 µm in both width and height. Due to the large detector size, the camera is coupled to the trinocular head (camera port) of the microscope without any magnification adjustments.

#### 2.1.3. Light Source

**Figure 1:**
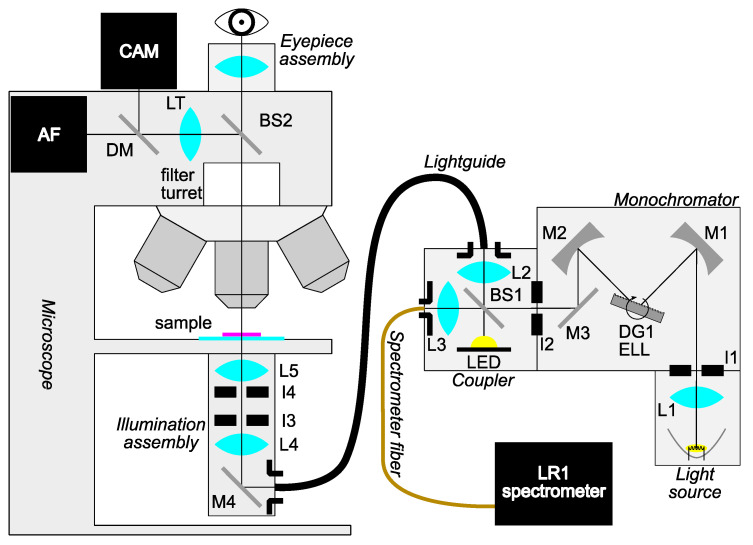
Outline of the hyperspectral microscope imaging (HMI) system setup. Light from the halogen lamp is collected by a lens and passed into a Czerny-Turner-type monochromator of custom design. Monochromatic light is then directed into the coupler, where part of the light is used for online monitoring via a spectrometer. The remaining part of the light, along with light from an optional high-power white LED used for orientation, is passed through a fiber bundle light guide. Using a custom Köhler illumination assembly, light impinges on the sample. Light from the sample is collected by an objective and passes through the microscope to either eyepieces or a camera used for data acquisition. Legend: Lx—lenses, Mx—mirrors, Ix—apertures, DG—diffraction grating, ELL—rotation stage, BS—beamsplitter, LT—tube lens, DM—dichroic mirror, CAM—camera, AF—autofocus system.

The main custom-made part of the system consists of a light source coupled to a specially designed illuminator for transillumination. Since spectral band selectivity is required, the filtered approach to hyperspectral imaging is implemented, in which only a handful of spectral bands can be selected for imaging to obtain relevant spectral features with the benefit of decreasing the acquisition time. Additionally, the spectral width of the illumination can be adjusted, and although this means decreasing the spectral resolution, it increases the signal-to-noise ratio at the same time while further decreasing acquisition time. Monochromatic light for sample illumination is obtained from a halogen lamp using a custom-made single-grating monochromator in the Czerny-Turner configuration [28]. The complete setup is schematically depicted in Figure 1. The monochromator, beam splitter, and condenser assembly with all other components of the HMI system are described in detail in Appendix A, while the system’s software development is outlined in Appendix B.

### 2.2. Validation and Calibration Protocols

**Figure 2:**
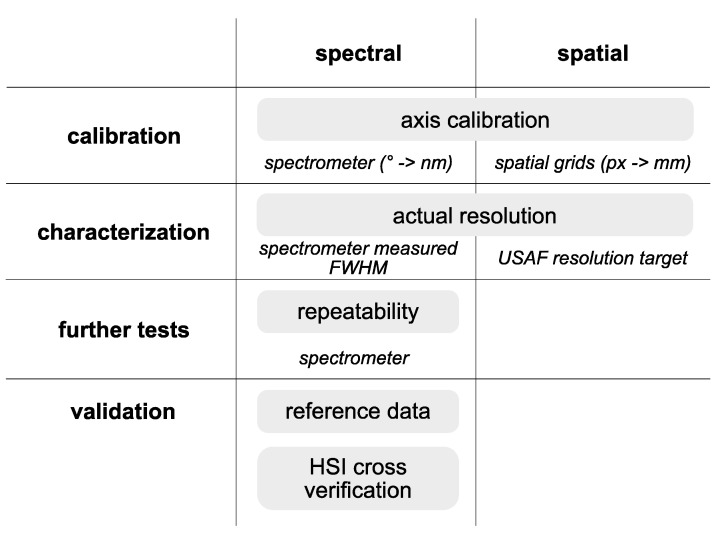
Protocol for calibration and validation of the hyperspectral microscope imaging (HMI) system. The core protocol is adapted from a general protocol proposed for spectral imaging systems with modifications specific to the monochromator-based imaging approach. Additionally, the validation step includes a comparison with a previously calibrated and validated hyperspectral imaging (HSI) system.

An important goal of this research was to implement a well-defined system design for validated quantitative imaging. Such design would facilitate system replication and, thus, allow its utilization in multicentric studies. The latter presents a key steppingstone in the process of development of quantitative biomarkers, an ultimate goal in any imaging modality development. To achieve these objectives, a protocol for calibration and validation of hyperspectral imaging systems [26] was adapted to suit the specifics of the hyperspectral microscopy setup and, more generally, to be compatible with an imaging protocol using light from a monochromator. A schematic overview of the protocol is shown in Figure 2.

### 2.3. Processing of Hyperspectral Images

Hyperspectral images consist of two spatial dimensions and a spectral dimension. This can be thought of as images containing a full spectrum in each pixel. Because such datasets are not intuitive for interpretation, appropriate methods must be used to extract interpretable information.

After acquiring the hyperspectral image $I^{acq.}\left[x,y,\lambda \right]$, an unobstructed beam reference $I^{ref}\left[x,y,\lambda \right]$ must be recorded. In our system, this was achieved by removing the sample and acquiring another image. Additionally, a dark reference $I^{dark}\left[x,\lambda \right]$ must be acquired if the dark current is not negligible. In the case of our system, due to the exceptionally low dark current and electronic noise, this measurement was not necessary. A white-reference-normalized transmittance image that mitigates problems of illumination homogeneity and nonconstant spectrum of the light source is calculated by dividing the acquired intensity image by the white reference image as

(1) $$I\left[x,y,\lambda \right]=\frac{I^{acq.}\left[x,y,\lambda \right]}{I^{ref}[x,y,\lambda ].}$$

For visualization purposes, an RGB image can be obtained from a hyperspectral image by projecting the normalized transmittance spectra to CIE XYZ tristimulus spectra [29], summing over all the wavelengths and dividing by the number of wavelengths. In this way, a CIE XYZ color space representation was obtained. A MATLAB function *xyz2rgb* was then used to convert to RGB color space appropriate for visualization, using the option of the standard D65 illuminant.

Furthermore, concentrations of stains, and more generally, endogenous and exogenous chromophores, can be extracted from the spectral data. Because the tissue slices in this study were thin, the contribution of light scattering was omitted, thus approximating the light-tissue interaction with a nonscattering form of the Beer–Lambert law [30]

(2) $$T\left(\lambda \right)=\frac{I}{I_{0}}=e^{-\mu_{a}\left(\lambda \right)z},$$

where T is the measured transmittance, I is the measured intensity, $I_{0}$ is the unobstructed beam reference, $\mu_{a}\left(\lambda \right)$ is the absorption coefficient, and z is the sample thickness. The absorption coefficient was estimated by combining the contributions of absorption coefficients of hematoxylin and eosin $\mu_{a,hematoxylin}$ and $\mu_{a,eosin}$ with their volume fractions in the shape of

(3) $$\mu_{a}\left(\lambda \right)=c_{h}\;\mu_{a,hematoxylin}\left(\lambda \right)+c_{e}\;\mu_{a,eosin}\left(\lambda \right).$$

Absorption coefficients were obtained from the literature [31] and were reported in relative units. They are, thus, applicable only for the generation of relative concentration maps. Sample thickness z was measured by focusing on the top and the bottom surfaces of the slide and reading out the focusing stage positions.

With the light-tissue interaction model set up, the concentrations of both histological dyes were extracted from measured transmittance spectra using the least squares fitting method *lsqnonlin* in MATLAB, with concentrations $c_{h}$ and $c_{e}\;$as the free parameters.

## 3. Results

### 3.1. Spectral Calibration and Characterization

**Figure 3:**
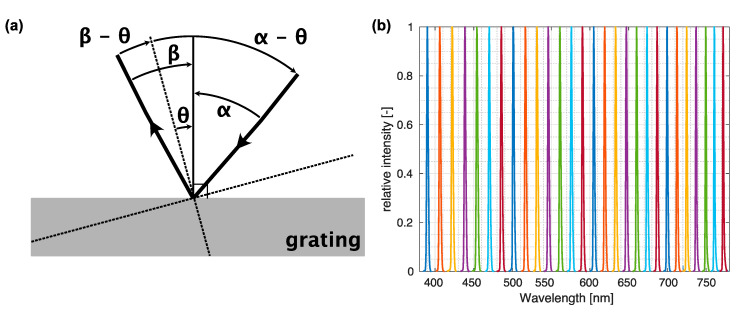
Monochromator angles and spectra. (**a**) Schematic of angles in monochromator equations. Arrows indicate the direction of positive angles. (**b**) Spectra acquired with the spectrometer (normalized to 1).

Multiple steps were needed to calibrate the system and test its performance. First, the wavelength scale was calibrated by monitoring the light emitted by the monochromator with a spectrometer, thus obtaining the reference central wavelength and spectral shape of the light. Light filtered by the monochromator was measured with an Ocean Optics USB2000+ UV-VIS spectrometer (Ocean Insight, Orlando, FL, USA) connected to the system instead of the ASEQ LR-1 used for online monitoring. The USB2000+ had a higher resolution than the LR-1 and was, thus, more appropriate for fine calibration of the system. Because only the relation of wavelength vs. monochromator absolute position in degrees and width of the spectrum was of interest, all measurements were normalized to 1, thus easing both visualization and processing. Although this step removes the information about the absolute intensity, it follows the image processing protocol standard to spectroscopy, where transmittance data is normalized to a reference intensity without a sample. A set of relative intensity spectra recorded in the range of −9° to −35° of the diffraction grating positions in steps of 1° corresponding to the visible light is presented in Figure 3b.

Calibration of the absolute monochromator position (expressed in degrees) to the peak wavelength of light emitted was based on the grating formula [32], accounting for the first order of diffraction, as

(4) λ=d[sin(α−θ)+sin(β−θ)].

**Figure 4:**
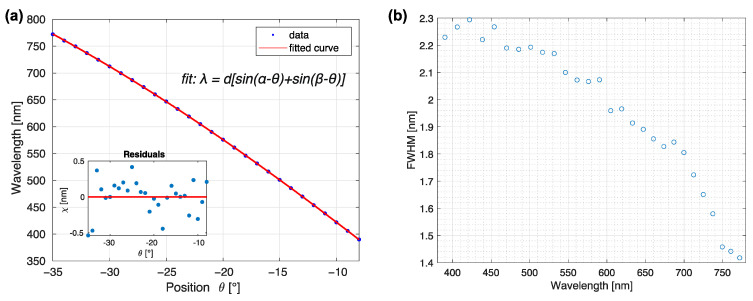
Monochromator calibration. (**a**) The relation between monochromator positions obtained from the rotation stage encoder and measured central wavelengths (blue dots) is calculated by fitting the grating formula (Equation (4), red line). (**b**) FWHM (full width at half maximum) as calculated from spectral measurements.

The grating constant d was specified by the manufacturer and set at 555.56 nm; the parameters α and β were angles of the incoming and diffracted light, respectively, and varied with the rotation stage position θ of the grating. Parameters α and β were computed by fitting the Equation (4) to the measured central wavelengths λ and corresponding stage positions θ. The definition of the parameters is given in Figure 3a. The results of the fit resulting in α=38.6 ± 0.1° and β=9.46 ± 0.1° are displayed in Figure 4a. Because the reliability of the calibration depends strongly on the precision of stage position reading, thorough testing of repeatability and reliability was performed later in this section as part of the characterization protocol.

Besides the calibration of the central wavelength, the spectral resolution was determined by evaluating the full width at half maximum (FWHM) of a given measured peak, shown in Figure 3b. The largest FWHM was around 400 nm (2.3 nm), which was taken as the system’s resolution; FWHM continually decreased with wavelength, reaching values of around 1.5 nm at the central wavelength of approximately 750 nm (Figure 3b). The observed FWHM trend is expected, considering the typical decrease of linear dispersion toward higher wavelengths [32].

**Figure 5:**
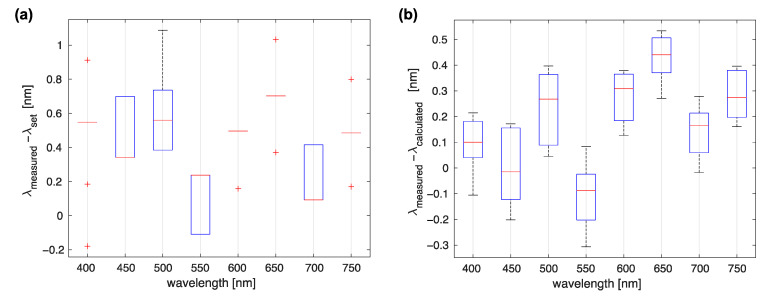
Monochromator wavelength selection repeatability. (**a**) Differences between the requested and spectrometer-measured wavelengths. (**b**) Differences between the wavelengths obtained from the calibrated encoder position compared to the spectroscopy results.

Next, the repeatability of the stage positioning was evaluated. Two possible ways to determine the central wavelengths of the light emitted from the monochromator were considered. First, a stage was sent to the required angle using Equation (4); this approach was simple but inaccurate because the precision of the stage was limited to 0.05°. A better option was to send the grating into an approximate position and then calculate the central wavelength from the included encoder that had an angular resolution of 0.0025°. The repeatability was tested by sending the stage to a set of wavelengths ten times, with homing initiated between measurements. After two repetitions for each measurement, the software was closed, and the stage disconnected from power to simulate different startups and evaluate encoder calibration effects. Values of the central wavelengths were recorded using the spectrometer and compared to the requested values as well as those calculated from the encoder positions. The results are presented in Figure 5.

These results show that due to the low positioning precision of the piezo resonant rotation stage, deviations between the true and the requested wavelengths were about 1 nm. By using the integrated encoder, differences lower than 0.5 nm were observed. This was also close to the resolution of the UV-VIS USB2000+ spectrometer, which was about 0.35 nm. Therefore, requested wavelengths were used only to label the slices in images, while for the processing and further analysis tasks, the wavelengths were calculated from the recorded stage positions. The spectral precision of our system was thus 0.5 nm, limited by the encoder resolution repeatability, and the resolution of 2.5 nm, limited by the spectral bandpass of the device.

### 3.2. Spatial Calibration and Characterization

After the spectral calibration, the spatial scale for each objective lens was evaluated to establish the size of the detector pixel image on the object plane, enabling accurate measurements of distances on the recorded images. This pixel size calibration was performed by acquiring images of the calibration stage micrometer R1L3S1P (Thorlabs, Newton, NJ, USA). Distance in µm in the object plane x was related to the size of the image on the detector n in pixels by a simple relation x=Δx·n, where ratio Δx must be calculated for each objective lens separately. The image inspection tool *imtool* in MATLAB was used to measure the distance in pixels on the image by using the length measurement tool and measuring between the edges of the dark stripes. These measurements were then used to calculate Δx based on the known distance between the lines. Spatial calibration revealed that at magnifications starting with the 10× objective lens, the pixel size in the image plane was below the diffraction limit, and thus, binning of the pixels can be implemented without loss in terms of resolvable features. The results of the spatial calibration are presented in Table 1.

Actual resolving power was also evaluated by the USAF1951 pattern R3L1S4P (Thorlabs, Newton, NJ, USA). The resolving power for the 5× objective lens was 181 line-pairs/mm, while the resolving power for other objective lenses was greater than 228 line-pairs/mm, which was the highest power available on the test target. Resolution for the 5× objective lens was, thus, (2.8 ± 0.4) µm, with the uncertainty given by the element resolution step of the USAF target. For the remaining lenses, the resolving power was estimated as the width of the edge in the USAF target and was (2 ± 0.1) µm for the 10× lens, (1.7 ± 0.1) µm for the 20× lens, and (1.3 ± 0.1) µm for both the 50× and the 100× lenses. Because the actual resolution in terms of lowest resolvable features was given by the quality of optical components and the wavelength of the light used for the imaging, the highest achievable resolution was determined to be 1.3 µm, and no increase in resolution was observed when using the 100× lens instead of the 50× one. Due to the trade-off between the resolution and FOV size, the 10× objective lens provides optimal resolution while still possessing a comparatively large FOV.

### 3.3. Validation

The system was validated by imaging a set of liquid dye samples sandwiched between two microscopy glasses. Samples were measured using a reference PerkinElmer Lambda 1050 UV/VIS/NIR (PerkinElmer, Waltham, MA, USA) laboratory spectrometer that served as the gold standard as well as our custom-made hyperspectral imaging (HSI) system [26,33].

The sample cells were prepared in a standard fashion for the microscopy of liquid samples as used in microrheology and microfluidics [34]. Briefly, the liquid diluted ink sample was placed by means of capillary action between a cleaned object and cover slides and sealed by means of a UV-cured optical adhesive (NOA63, Norland Products, Jamesburg, NJ, USA) to create a channel for the ink. The thickness of the sample cells, as measured by the microscope, was approximately 100 µm with deviations across the sample cells of about 20%. In the resulting cell samples, the amount of the pigment varied across the sample due to two factors: (i) variation in the cell thickness and (ii) separation of the pigment in ink from the solvent.

For the reference measurement of sample cells, the reference laboratory spectrometer with a 3D WB detector module was used. For validation, blue and red ink were employed because their respective spectra were not overlapping. Spectra were measured in the collimated transmittance mode and normalized to an unobstructed beam.

**Figure 6:**
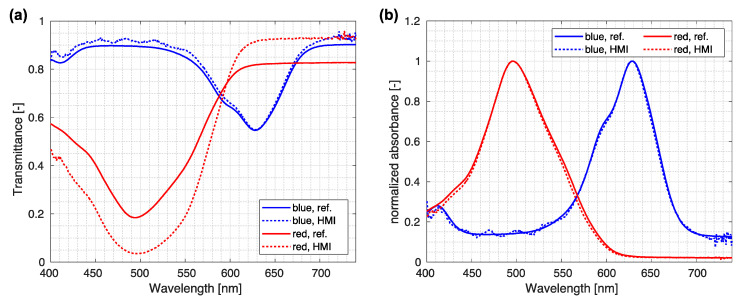
Comparison between spectra measured by HMI and reference spectra. (**a**) Comparison of raw normalized spectra acquired using the reference spectrometer and custom-made HMI system. (**b**) Normalized absorbance from reference and HMI measured spectra.

The ink cells were imaged using our custom-made HMI system. Spectra were normalized, and an averaged normalized spectrum from the central part of the sample was used for comparison. First, the raw normalized spectra were compared for both the HMI system and reference spectrometer (Figure 6). As the agreement of absolute spectra was expected to be inadequate, normalized absorbances were calculated by taking the logarithm of the transmittance value *T* and normalizing it to the maximal value,

(5) $$\tilde{A}\left(\lambda \right)=\frac{ln\left(T\left(\lambda \right)a\right)}{max[ln\left(T\left(\lambda \right)a\right)]},$$

where a was the normalization factor considering that different modalities collected different amounts of light. Values of the normalization factors a were 1.17 for red ink and 1.03 for blue ink. Figure 6 shows an excellent agreement between the reference and the HMI systems when considering normalized absorbance.

**Figure 7:**
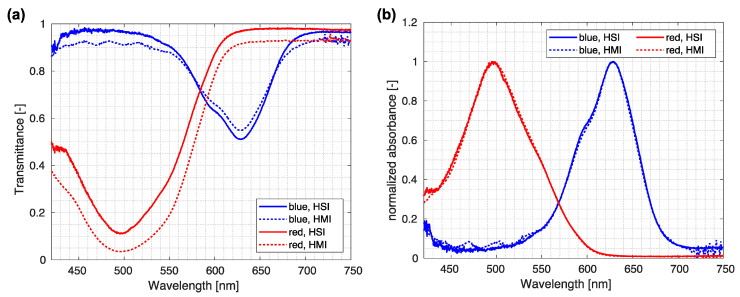
Comparison between HMI and HSI spectra. (**a**) Direct comparison of spectra acquired using custom-made HMI and HSI systems. (**b**) Normalized absorbance spectra obtained by custom-made HMI and HSI systems.

In addition, we compared the HMI spectra with those obtained by the custom-made HSI system for macroscopic samples [26,33]. Comparison between both modalities, shown in Figure 7, reveals that normalized raw spectra exhibit similar shapes, while normalized absorbance values demonstrate almost perfect agreement. An additional indicator of an excellent agreement between both modalities is the same normalization factor *a* = 1.05, obtained for red as well as blue ink.

### 3.4. HMI of a Tissue Sample

**Figure 8:**
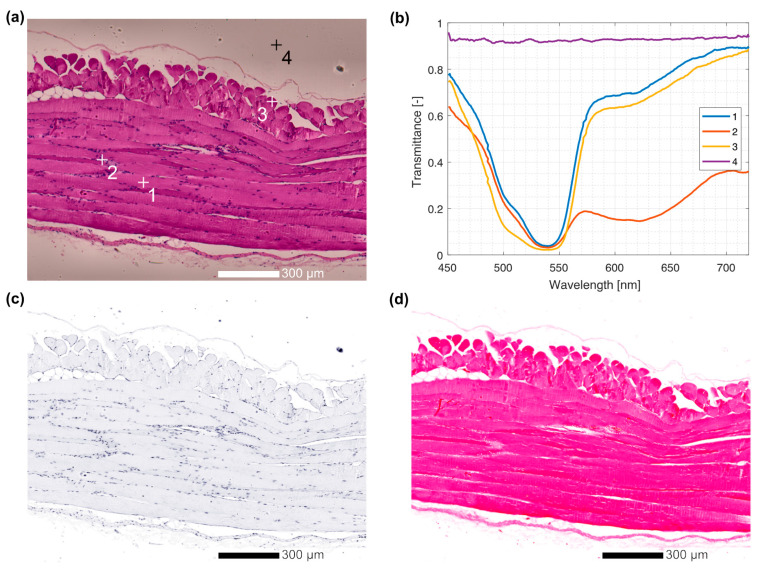
A hematoxylin and eosin-stained histology slide of a healthy murine abdominal wall at 10× magnification recorded by HMI. (**a**). An RGB image reconstructed from the HMI data. Spectra were selected at different image points and marked by + signs and labeled by numbers. (**b**). Spectra corresponding to the marked image points. Based on the spectra, individual hematoxylin and eosin maps were calculated using a Beer–Lamber attenuation law and presented in false color ((**c**)—hematoxylin, (**d**)—eosin).

To demonstrate the use of the HMI system, we imaged a standard hematoxylin and eosin-stained histological sample of the murine abdominal wall [35,36]. The raw spectral data were first normalized by an unobstructed light field, thus normalizing the values to transmittance as well as eliminating illumination field inhomogeneity. Figure 8a shows an RGB projection of a hyperspectral cube obtained using the methods described in Section 2.3. This image is analogous to a classical RGB histology image; in fact, it appears closely similar to a standard histology image before background removal and contrast enhancement in postprocessing. Figure 8b shows spectra selected from the image shown in Figure 8a at three different points on the sample itself and at one point on a section containing only glass without tissues.

The point labeled 2 is selected from a section of the sample subsided by cell nuclei. The nuclei are stained strongly by the hematoxylin dye that is blue-black in appearance. The spectrum at this point reveals a high absorption and, hence, lower transmittance throughout the visible part of the spectrum. Points annotated 1 and 3 are in the cell cytosol and are thus primarily stained by eosin. Eosin is a dye that is purple-red in color. Consequently, a large absorption in the green part of the spectrum, roughly between 500 nm and 550 nm, is present.

A marked difference between the intensity of color at points 2 and 3 is visible in the RGB projection (Figure 8a). This difference is even easier to discern in the transmittance spectra as a decrease in the fraction of the transmitted light resulting from the increased absorption due to different concentrations of the stain. The point denoted 4 corresponds to a part of the slide where no sample is present. At this point, the transmission spectrum does not contain any significant features. The small oscillations attributed to interference between optical surfaces are due to the presence of the object slide, embedding resin, and cover slip.

Following the qualitative observations, a more quantitative approach was applied to the images to extract hematoxylin and eosin maps, as described in Section 2.3. Using this approach, it was possible to construct false color relative stain concentration maps shown in Figure 8c,d. The applicability of such stain concentration maps for further processing is vast. Possible applications that exploit the wealth of information contained therein include, among others, analysis of the chemical environment influencing the binding affinity of stains as well as image analysis algorithms for digital histology on separate nuclear and cellular images.

## 4. Discussion

The calibration and validation protocols can be, with different rigor, generalized to other existing HMI systems. Spatial calibration should be, in general, similar for all systems, be it a point-scanned whiskbroom or a rapid, albeit spectrally limited, snapshot imager. It is important to note that, regardless of the methodology used, calibration of the spectral resolution and step size must be performed. In a case such as the one presented in this paper, where excitation scanning is used, the proposed spectrometer measurements should be sufficient. However, when using an emission-scanning filtered methodology of acquisition, a previously calibrated and verified source of monochromatic light needs to be used to evaluate the resolution, spectral broadening, and spectral step.

The validation results for the liquid ink samples were compared with both the reference laboratory spectrometer as well as with our custom-developed laboratory hyperspectral imaging (HSI) system for macroscopic samples. Such two-fold validation has many advantages. First, it demonstrates the reliability of the system and, thus, sets the foundation to perform quantitative imaging. Second, the cross-validation with the HSI system enables analysis and comparison of spectral imaging data on two dramatically different length scales and could, thus, provide a channel to establish a better connection between the effects of microscopic tissue changes on macroscopically observed optical properties in the future.

Carefully evaluating the system performance and determining its repeatability in terms of central wavelength and proper determination of spectral resolution is of key import. If a system is characterized in such a way, the results are consistent, and the repeatability of experiments conducted using such a device is ensured.

As an illustration of the possible use of the developed system, we presented a hyperspectral image of a histology slide. The spectral information contained within the image is successfully used to decouple the contributions of hematoxylin and eosin stains. Although the results presented are preliminary and, as such, not clinically significant, the potential to study the staining of histology samples quantitatively is promising and could prove a useful tool in the future development of quantitative histology.

The great power of hyperspectral imaging lies in its ability to discern small changes in different chromophore concentrations that reflect the changes in the surrounding tissue. Using the system presented in this paper, two different approaches can be envisioned to evaluate these changes. First, tissue-native chromophores, such as hemoglobin, collagen, and cytochromes, could be evaluated through their specific absorption spectra. This approach would, however, require unstained tissue samples and in-depth knowledge of the effects tissue fixation and embedding have on their optical properties. Alternatively, the chemical environment of the tissue also affects the staining process. Thus, observation of small changes in histology stain concentration could reveal processes in the tissue that would otherwise remain unnoticed.

In this paper, we have shown a decomposition of an H&E histology slide into individual hematoxylin and eosin maps using hyperspectral methods. Such a deconvolution into individual stain images is a common step in quantitative histology evaluation [37]. It can be achieved by approaches generally termed color deconvolution that includes the calculation of stain-specific weighted averages of RGB channels in a normal color image [37]. More advanced methods could also be used, such as color phasors [38]. Due to the limited information contained in a normal RGB image, only a handful of different stains can be evaluated, and analysis depends on the quality of the illumination. In hyperspectral imaging, information about sample staining is contained in hundreds of different channels, thoroughly describing the sample absorbance. This, in turn, enables spectroscopic analysis that can identify different stains even when their absorption spectra are similar or overlapping.

A great advantage of our system is the modularity that is inherent to its design. The modularity and versatility of the presented system may help in the development of new imaging protocols for specialized samples where existing commercial systems offer insufficient adaptability.

## 5. Conclusions

In this paper, we have shown the calibration, characterization, and validation of a custom-based HMI system for biomedical applications. To the best of our knowledge, this is the first HMI system that was cross verified with an HSI system for macroscopic samples. This validation step enables imaging of the same samples at dramatically different magnifications, making possible the quantitative comparison of optical as well as morphological properties on different scales.

To demonstrate the system performance, a histology slide stained with hematoxylin and eosin was imaged, and hyperspectral image processing methods were used to first represent it in an RGB projection as well as to construct relative stain concentration maps.

The primary objective of the study, the validation of the system following protocols, was achieved through the implementation of the modified calibration and validation protocol. Such protocols are instrumental in the standardization of HMI systems and a necessary steppingstone in establishing HMI as a diagnostic tool.

The standardization of HMI systems is of utmost importance in order to advance this new imaging modality and to meet future diagnostic challenges. HMI in biomedicine is still in its infancy, and our work is an attempt to improve technical standards while keeping in mind engineers, physicists, and clinicians alike.

## Figures and Tables

**Table 1:** Distance calibration of microscope objective lenses. Distance on the ruler *x* that spans *n* pixels for each objective lens gives the size of the pixel ∆*x* on the image plane.

Objective Lens	*x* [μm]	*n* [px]	Δ*x* [μm]
5×	2000	1530 ± 10	1.307 ± 0.009
10×	1000	1530 ± 10	0.654 ± 0.005
20×	500	1510 ± 10	0.331 ± 0.002
50×	250	1894 ± 10	0.132 ± 0.001
100×	100	1541 ± 10	0.065 ± 0.001

**Figure A1:**
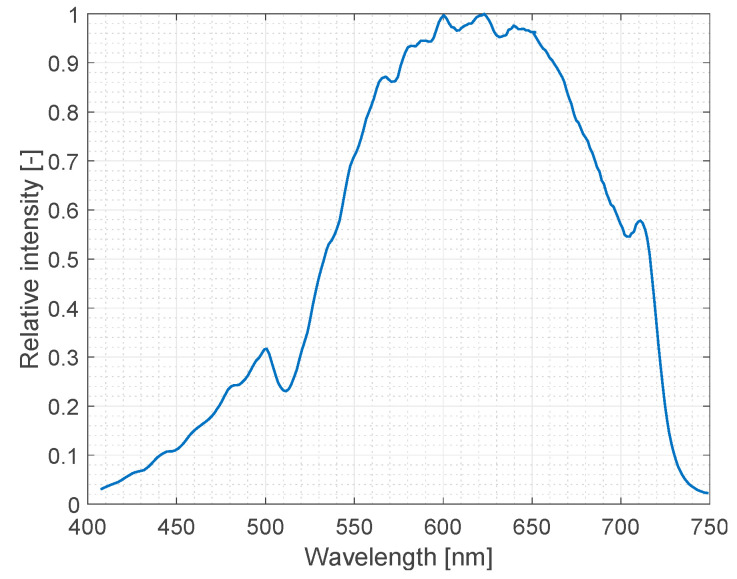
Relative intensity of the monochromator light source as detected by the camera. The intensity was normalized to the maximum value to aid with visualization. Please note that the intensity recorded by the camera is a product of the halogen light source spectrum, transmittances of all the optical components in the light path, and sensitivity of the detector.

**Figure A2:**
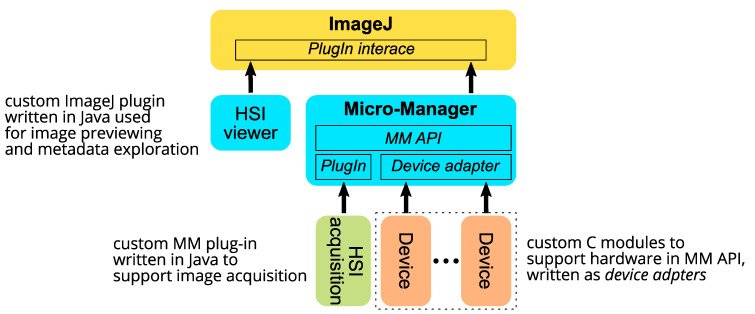
The software architecture of the control system. The main framework is ImageJ, which runs either a custom HSI viewer plugin written in Java or the Micro-Manager (MM) environment. To communicate with Micro-Manager API, each device has a C class that implements the Micro-Manager device adapter interface, communicating with Micro-Manager on one side and the device on the other. For acquisition control, a separate plugin written in Java implementing Micro-Manager plugin architecture is used. This plugin can access all the devices in the configured system via the general Micro-Manager API.

## Data Availability

The data that support the findings of this study are available upon reasonable request from the authors.

## Appendix A

### Appendix A.1. Monochromator

We will describe the system by following the pathway of the light. First, the light from a halogen source enters the adjustable entrance slit I1 (Thorlabs, Newton, NJ, USA) of the monochromator, which is positioned in the focus point of a silver-plated concave mirror M1 (CM508-200- P01, Thorlabs, Newton, NJ, USA) with a focal distance of 200 mm, so that the image of the slit is formed at infinity. For better coupling, the light from the lamp is first collected by a condenser lens L1, while the collimated light beam then impinges on a ruled reflective diffraction grating with 1800 lines/mm G1 (GR50-1850 Thorlabs, Newton, NJ, USA). The diffracted light is then focused and redirected using a second identical concave mirror M2 forming a spectrally separated image of the entrance slit on the exit slit I2. To change the wavelength selected by the exit slit of the monochromator, grating G1 is rotated by a computer-controlled piezo rotation stage ELL (Elliptec ELL18K/M, Thorlabs, Newton, NJ, USA). The slits are adjusted to achieve the best ratio of light throughput and spectral full width at half maximum (FWHM). For better positioning of the output port, the exiting light is redirected by 90° to the side of the monochromator by means of an ordinary silver-plated mirror M3.

### Appendix A.2. Beam Splitter

After the monochromator, light is coupled into a cage system with a built-in 90:10 beam splitter BS1. There, 90% of light is collected using a lens L2 into a fiber bundle light guide with a core diameter of 6 mm to be used for sample illumination, while 10% is coupled using a collector lens L3 into an optical fiber that is connected to an LR1 spectrometer (ASEQ, Montreal, QC, Canada). This light serves the purpose of online monitoring of the light used for sample illumination. Additionally, to locate regions of interest that should be imaged on the sample, a high-intensity white LED is placed so that 10% of its output is also coupled into the light guide and can be used for sample illumination.

### Appendix A.3. Condenser Assembly

Illumination of the sample with the light from the light guide is achieved by a custom-made condenser assembly designed to provide a homogeneous illumination of the sample by means of the Köhler illumination [39]. First, light from the light guide is redirected using a mirror M4 and focused into the plane of the condenser’s diaphragm I3 by a collector lens L4. Condenser lens L5 is then aligned so that it creates an image of the field diaphragm I4 on the sample plane while simultaneously creating an image of the condenser’s diaphragm at infinity. This ensures that the sample conjugate planes are separated from the source conjugate planes, and that illumination is homogeneous. The illuminated area of the sample is selected by adjusting the field diaphragm, while the condenser’s diaphragm regulates the numerical aperture of the light cone impinging on the sample.

### Appendix A.4. Control Modules and Power Supply Units

To control the whole system, several modules and power supply units are included. In addition to the standard motion controls described at the beginning of this section, three encoders with digitizers are added to the system. An ND221B digitizer connected to an MT25B encoder (Heidenhain, Traunreut, Germany) provides information on the z-axis position with a precision of 0.5 µm; for the *x-* and *y*-axis, two TGM 130 encoders with an NP-21 two channel digitizer (Iskra Tela, Ljubljana, Slovenia) provide lateral position with 1 µm precision. All the positioning information is collected by a computer using an RS-232 serial connection. A laboratory power supply, which could also be controlled by a PC, is used to power the 240-W, 12-V halogen lamp in the monochromator. Switching the halogen lamp and the LED is performed by a custom-made control box incorporating the switching electronics and an adjustable current source based on the LM317 integrated circuit [40] to power the white LED.

### Appendix A.5. Other Components

Before reaching the camera, light passes through an infinity-corrected microscope objective lens mounted on a motorized objective revolver; the light then travels through a reflector turret, where an additional filter could be added, if needed. After that, the output of the trinocular head is selected by a 90:10 beamsplitter BS2 so that 90% of the light is directed towards an eyepiece assembly or through a tube lens LT into the camera. A dichroic mirror DM is built into the microscope head to allow for a triangulation autofocus system based on an IR laser, which sets the upper limit of the hyperspectral imaging at 750 nm. The position of the camera on the microscope head is adjusted by means of an adjustable c-mount extension tube so that parfocality with the eyepieces is achieved.

Additional modules, such as an RGB camera for online monitoring, could be attached to the binocular ports by means of an eyepiece to the SM1 threaded adapter (Thorlabs, Newton, NJ, USA).

### Appendix A.6. Resulting Spectrum

After the light from the source passes the components described earlier in this section and finally reaches the camera, its intensity is a product of the light source spectrum, transmittances of all the components (such as beam splitters, dichroic mirrors, and lenses), and quantum efficiency of the camera. To eliminate these effects, the acquired spectra are usually represented in terms of transmittance; the actual raw values are normalized (divided) by an unobstructed beam spectrum. The study of raw white reference spectra used for this normalization can, however, aid in determining the useful spectral range of the system. To illustrate this, Figure A1 depicts an example white reference acquired using the camera over the whole usable range of the system. It can be seen that the recorded intensity decreases at the edges due to the effects of detector sensitivity and limited transmittance of visible light-optimized optical components within the microscope. Irregular features in the spectrum are a consequence of beam splitter and dichroic mirror transmittance. The plot in Figure A1 clearly illustrates the usable imaging range between 420 nm and 750 nm.

## Appendix B

Because the system is completely modular and could be easily modified, the control software should fulfill the following requirements:

1. Software should be modular and available for source code customization.
2. Software should support (I) a plug-in-based architecture for different microscopy modules as well as (II) a simple yet general programming interface for the addition of new modules.
3. Software should support as many of the integrated components as possible out of the box without custom-made drivers.
4. Since the acquisition of larger samples will be implemented in the future, software should support the scripted acquisition and whole slide scanning.

A popular software in the field of microscopy that is often used to control both general production and custom microscopes is an advanced modular microscopy control framework Micro-Manager [41], built atop the image processing toolbox ImageJ [42,43], which already supports the Zyla 5.5 camera used in our system. It offers its own scripting language for automated acquisition and a series of plugins that allow for whole slide scanning. Additionally, it supports generalized component types, which can be used to integrate any component not supported out-of-the-box in the software through an intermediate interface. As it is built on top of ImageJ, it already supports many different image formats and image analysis tools, and due to this modularity and high customizability, it is the optimal framework that fulfills the requirements above. The design of the software and its subsequent integration into the Micro-Manager and ImageJ environment consists roughly of two parts (device adapters implementing hardware functions written in C programming language and plugins that control the system written in Java programming language), as depicted schematically in the diagram of the software implementation (Figure A2).
